# Clinical characteristics and prognoses of six patients with multicentric giant cell tumor of the bone

**DOI:** 10.18632/oncotarget.13057

**Published:** 2016-11-03

**Authors:** Chenglei Liu, Yawen Tang, Mei Li, Qiong Jiao, Huizhen Zhang, Qingcheng Yang, Weiwu Yao

**Affiliations:** ^1^ Department of Radiology, Shanghai Jiao Tong University Affiliated Sixth People's Hospital, Shanghai, China; ^2^ Department of Pathology, Shanghai Jiao Tong University Affiliated Sixth People's Hospital, Shanghai, China; ^3^ Department of Orthopedics, Shanghai Jiao Tong University Affiliated Sixth People's Hospital, Shanghai, China

**Keywords:** giant cell tumor, multicentric, bone imaging

## Abstract

Multicentric giant cell tumor of the bone (MGCT) is a rare entity whose radiographic, pathological and biological features remain confusing. We retrospectively reviewed six patients (1 male, 5 female; average age, 22.33 years) treated for confirmed MGCT between 2001 and 2015. The patients' clinical information, images from radiographs (*n* = 14), CT (*n* = 13), MRI (*n* = 8), bone scintigraphy (*n* = 1) and PET-CT (*n* = 2), as well as histologic features, treatment and prognosis were analyzed. A total of 17 lesions were detected: 4 around the knee joint, 3 in the greater trochanter and head of the femur, 5 in the small bones of the feet, and 2 in flat bones. All these lesions occurred in an ipsilateral extremity. One patient had Paget's disease. On radiographs and CT, 12 lesions exhibited sclerotic margins or patchy sclerosis, 8 showed cortical discontinuity, and 5 showed soft tissue masses. On histopathology, 8 lesions showed signs of sarcomatous transformation and one had transformed into osteosarcoma. Ten lesions in 4 patients were initially treated with surgery, and 3 showed local recurrence. Seven lesions in 3 patients were treated with denosumab. All the patients are currently stable without metastasis. These results suggest MGCT tends to occur in uncommon sites with sclerosis. Because these lesions can be aggressive, patients should be carefully monitored for the recurrence or formation of other lesions, especially in an ipsilateral extremity.

## INTRODUCTION

Giant cell tumors of the bone (GCTs) are relatively common, benign, aggressive lesions that represent approximately 20% of all benign bone tumors and approximately 5% of primary bone tumors [1]. Based on the 2013 WHO classification of tumors of the bone and soft tissue, GCTs are classified as exhibiting intermediate (rarely metastasized) or malignant biological behavior [2] with frequent local recurrence after surgical treatment, lung metastasis, and latent malignant transformation [3]. Radiologically, the classical appearance is an expansible, eccentric geographic lucent lesion without sclerotic margins. Pathologically, GCT is composed of multinucleated, osteoclast-like giant cells, monocytic round macrophage-like cells, and spindle-shaped fibroblast-like stromal cells [4]. Its pathogenesis was reportedly closely associated with the RANK pathway because osteoclast-like giant cells and their precursors express receptor activator of nuclear factor kappa B (RANK), while mononuclear stromal cells express RANK ligand (RANKL), which are key mediators of osteoclastic activation [5]. Usually, GCT occurs as a solitary lesion at the end of a long tubular bone. When multiple lesions are encountered in a single case, they are defined as multicentric giant cell tumor of the bone (MGCT) [6]. MGCT is exceedingly unusual, occurring in less than 1% of cases of GCT [7, 8]. The occurrence of MGCT was classified as synchronous or metachronous by Hoch [9]. At present, surgical resection remains the mainstay of treatment for GCT [10]. In cases of MGCT, however, surgical resection may be impossible and can lead to severe morbidity. Therefore, medical treatment has been proposed as an adjunctive therapy to reduce osteoclast activity [3]. Recent studies have suggested that denosumab may be a potentially promising adjuvant therapy [11, 12]. Denosumab is a fully human monoclonal antibody that inhibits RANKL, thereby preventing RANK-RANKL interaction and inhibiting the maturation of osteoclasts [3].

Several studies of MGCT reported in the literature, but most were single cases or small series; discussions of more than five cases are rare [6, 11, 13–20]. At present various issues related to the radiographic and pathological features of MGCT and its biological behavior remain unresolved. Dhillon et al. noted that MGCT was not a more clinically aggressive form than solitary GCT, and was radiologically and pathologically indistinguishable from solitary GCT [8]. However, there have been reports that MGCT lesions were more locally aggressive than their solitary counterparts [15, 21], as well as having uncommon radiological features [9].

Although previous reports have demonstrated the potential efficacy of denosumab for the treatment of GCT [5, 22], there have been no studies of the efficacy of denosumab in cases of MGCT. Here, we discuss the clinical characteristics, radiographic features, pathological analyses and prognoses of six patients with MGCT. In addition, we compared the radiographic features of three MGCT patients before and after treatment with denosumab.

## RESULTS

### Patients

**Figure 1:**
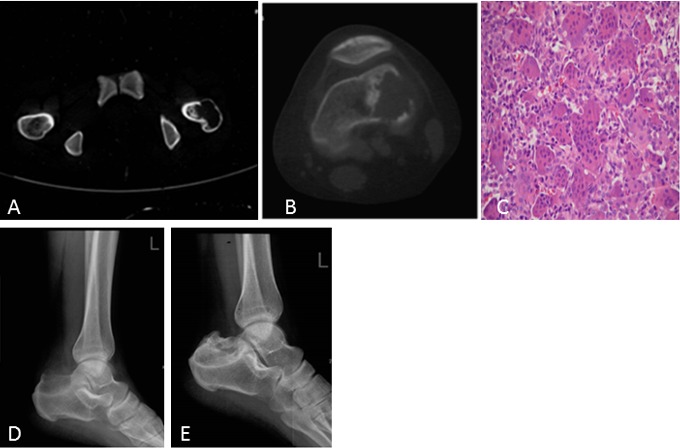
A 19-year-old woman with pathologic fracture of the greater trochanter of the left femur **A.** CT shows an osteolytic lesion with cortical discontinuity and patchy sclerosis. **B.** Eight years after initial presentation, knee CT shows another lesion in the distal femur. **C.** Microscopic pathology, HE staining at 200X at the lesion of the distal femur. The specimen showed the histologic appearance of a giant cell tumor of the bone, but proliferative active mononuclear cells, pathological mitosis and immature osteoid tissue were seen in some areas. **D.** Four years later after the second lesion occurred, a lateral radiograph of the calcaneus showed an osteolytic lesion. **E.** One year after treatment with the RANKL inhibitor denosumab, a lateral radiograph of the calcaneus showed increased sclerosis and cortical bone reformation.

**Figure 2:**
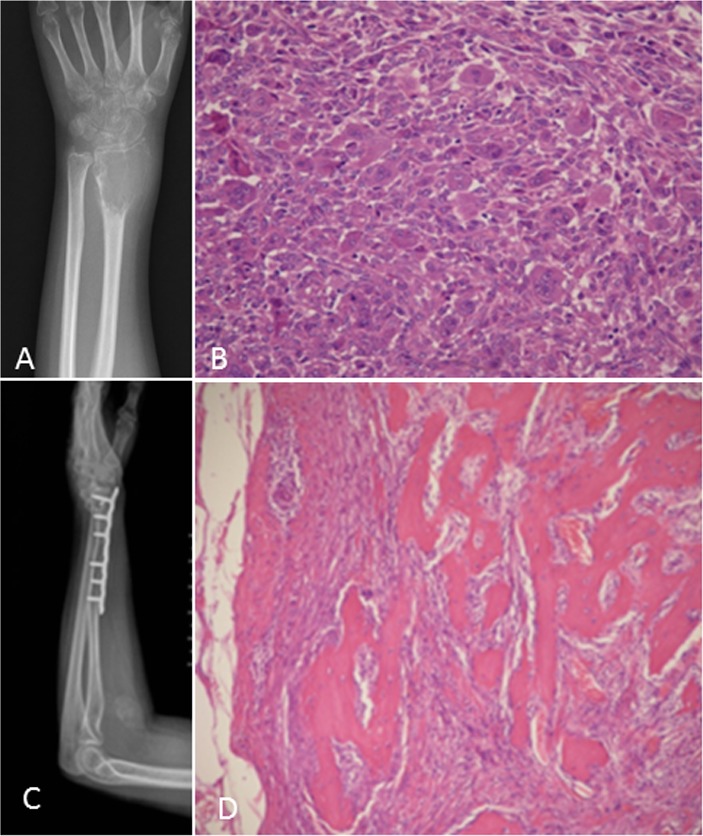
A 29-year-old woman with swelling and stiffness of the left distal radius **A.** Postero-anterior radiograph shows a large expansile subarticular osteolytic lesion; cortical incomplete. **B.** Histologic appearance of a giant cell tumor of the bone, stromal cell active proliferation, frequent mitotic figures and pathological mitosis. (HE 100X). **C.** Two years after treatment with curettage and internal fixation, another lesion occurred in the forearm. **D.** Pathological section shows giant cell tumor of the bone with malignant transformation consisting of well-differentiated osteosarcoma (HE 200X).

**Figure 3:**
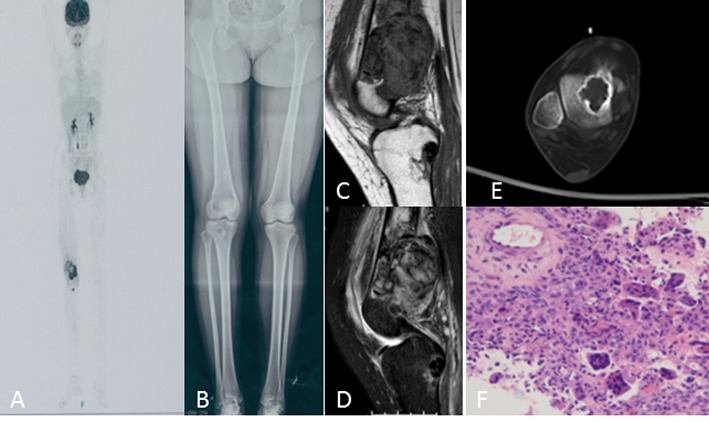
A 20-year-old woman with right knee swelling and pain **A.** PET/CT shows right distal femur, proximal tibia, femur head, and talus with increased intake. **B.** Antero-posterior radiograph of the entire lower extremity shows multifocal osteolytic lesions. **C.**, **D.** Knee MRI shows irregular, poorly defined soft tissue with T1 intermediate and T2 heterogeneous hyperintensity, extensive soft tissue edema in the distal femur and T1 hypointensity and T2 inhomogeneous low signal intensity in the proximal tibia. **E.** Talus CT shows osteolytic lesions with sclerotic ring margin. **F.** Histologic appearance of the giant cell tumor of the bone.

**Figure 4:**
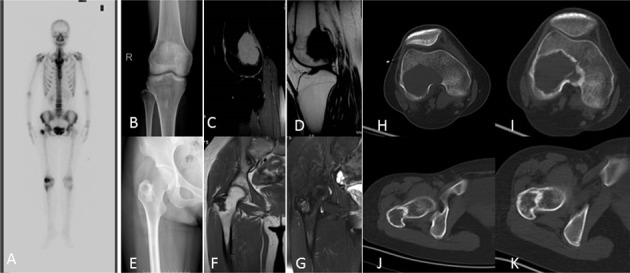
A 27-year-old woman with right knee pain **A.** Bone scintigraphy showed a diffuse increased radionuclide uptake in the greater trochanter of femur, and a peripherally increased uptake and photopenia centrally in the distal femur. **B.** Anteroposterior radiograph of the knee shows a well-circumstanced osteolytic lesion with a narrow zone of transition. **C.**, **D.** MRI shows a well-defined lesion with T2/T1 homogeneous intermediate and hyperintensity. **E.** Antero-posterior radiograph of the pelvic shows an osteolytic lesion with a sclerotic ring margin in the greater trochanter of the femur. **F.**, **G.** MRI shows an irregular lesion with T1 low signal intensity and T2 heterogeneous hyperintensity. **H.**, **I.** CT shows ring-like sclerosis around the outer margin after four months of treatment with denosumab. **J.**, **K.** CT shows increased sclerosis in the center of the lesion.

**Figure 5:**
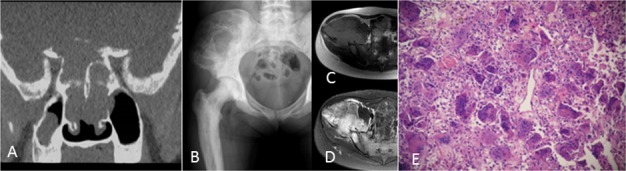
A 21-year-old woman with Paget's disease **A.** Coronal reconstruction CT of the basicranial bone shows osteolytic destruction with soft tissue mass. **B.** Anteroposterior radiograph of the pelvis shows a large multilocular hypodense lesion at the iliac bone. **C.**, **D.** MRI shows T1 intermediate intensity and T2 heterogeneous hyperintensity with a low stipe signal. E: Histologic appearance of a giant cell tumor of the bone at the iliac bone.

**Figure 6:**
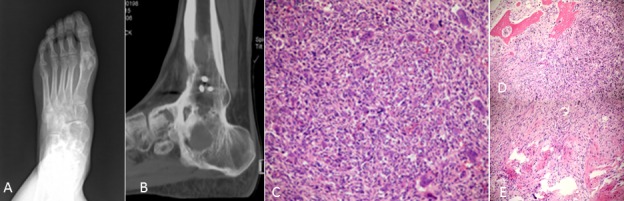
An 18-year-old man presented with pain, swelling and activity limitation **A.** Eight years after treatment, radiograph showed poorly circumstanced osteolytic lesions of the left navicular bone of foot and talus. **B.** Sagittal reconstruction CT shows multifocal osteolytic lesions. **C.**-**E.** Pathological section showed decreased giant cells with rich proliferative active spindle cells, reactive bone hyperplasia and massive fibrous tissue proliferation.

The clinical data of the MGCT cases are summarized in Table 1. This study consisted of 5 women and 1 man. The patients' ages upon initial presentation ranged from 18 to 29 years, and the mean age was 22.33±4.54 years; 3 patients were younger than twenty years old, and the oldest patient was only 29 years old. 3 cases were metachronous (Figure 1, 2, 6), and 3 cases were synchronicity (Figure 3, 4, 5). A total of 17 lesions were detected in 6 patients, among whom 2 patients had 4 tumors, 1 patient had 3 tumors, and 3 patients had 2 tumors. One case was associated with Paget's disease. The initial main complaints of the patients included intermittent dull bone pain dominating the scenario (4 cases), swelling (3 cases), decreased joint motion (1 case) and pathological fracture (1 case). Three cases underwent initial ALP test, 2 cases were performed ALP, LDH and calcium, magnesium, phosphorus test, and 1 case underwent ALP and calcium, magnesium phosphorus test. Only one case was accompanied by hypophosphatemia (Figure 5), the others were in normal levels.

**Table 1:** Clinical features of multi-centric giant cell tumors of the bone

Case	Age	Sex	Date	Bone involvement	Symptoms	Initial blood tests	Therapy
1	19	F	June 2001 Dec 2005 May 2009 Dec 2014	Greater trochanter of left femur recurrence Left distal femur Left calcaneus	Pathological fracture	ALP 85U/L	Curettage and cement placement Curettage and cement placement Curettage and bone grafting denosumab
2	29	F	July 2013 Feb 2015	Left distal radius Left distal radius recurrence Left forearm	swelling and stiffness	ALP 27U/L	Curettage and internal fixation Surgical excision Surgical excision
3	20	F	June 2015	Right distal femur Right proximal tibia Right femur head Right talus	Swelling, pain	ALP 66U/L; LDH 140U/L Calcium 2.32mmol/L Magnesium 0.92mmol/L Phosphorus 1.25mmol/L	denosumab
4	27	F	July 2015	Right distal femur Greater trochanter of left femur	pain	ALP 75U/L; LDH 170U/L, Calcium 2.42mmol/L, Magnesium 0.99mmol/L, Phosphorus 1.07mmol/L	denosumab
5	21	F	Feb2015	Right iliac bone Basicranial bone	intermittent pain	ALP 60U/L Calcium 2.63mmol/L Magnesium 0.95mmol/L Phosphorus 0.83mmol/L	Surgical resection and internal fixation Surgical resection
6	18	M	July 2007 April 2015	Left distal tibia Left distal tibia recurrence Left navicular bone, talus, calcaneus	pain, swelling	ALP 68U/L	Curettage and internal fixation amputation

Abbrviations: F=female, M=male, ALP= alkaline phosphatase, LDH= Lactate dehydrogenase.

### Location

The involved sites were the femur head (1 lesion), greater trochanter of the femur (2 lesions), distal femur (3 lesions), calcaneus (2 lesions), proximate tibia (1 lesion), distal tibia (1 lesion), distal radius (1 lesion), forearm (1 lesion), iliac bone (1 lesion), basicranial bone (1 lesion), navicular bone (1 lesion) and talus (2 lesions). Nine lesions were located in long tubular bones, among which 4 lesions were around the knee joint, and 3 lesions were located in the greater trochanter and head of the femur. Moreover, 5 lesions were located in the small bones of the feet, and 2 lesions were situated in flat bones. More interestingly, all of the lesions of each patient occurred in the ipsilateral extremity, and no tumors were detected in the contralateral position.

### Treatments

The treatment selection for MGCT is summarized in Table 1. Ten lesions in 4 patients were initially treated with surgery. Two patients did not undergo surgery. Seven lesions in 3 patients were treated with denosumab (120 mg, hypodermic injection) at 4-week intervals for a total of six times, vitamin D (≥400 IU) and calcium (≥600 mg) supplementation daily. No patients were treated with radiation.

### Imaging characteristics

On radiographs (*n* = 14), 16 lesions manifested as geographic lucent lesions, and 1 lesion showed soft tissue density with stripe-like calcification (Figure 2B). Among these lesions, 9 lesions showed ring-like sclerosis (Figure 3B), 3 lesions showed patchy sclerosis (Figure 4E), and 4 lesions showed narrow transition without sclerosis (Figure 4B). Based on Campanacci et al's grading system[23], 5 lesions were classified as grade III, 10 lesions as grade II, and 1 lesion as grade I. One lesion located in the calcaneus was treated with denosumab after one year, and a radiograph demonstrated shrinkage of the osteolytic zone and the formation of sclerosis in the center of the lesion and adjoining bone cortex (Figure 1D-1E). Four lesions showed increased sclerosis in the center and outer margin after six months.

On CT scanning (*n* = 13), 13 lesions showed marrow replacement by tissue with homogeneous attenuation, with homogeneity in 1 lesion. Nine lesions more clearly demonstrated sclerotic margins or patchy sclerosis (Figure 1A-1B). Three lesions showed a narrow transition without sclerosis. Eight lesions showed cortical discontinuity, and 5 lesions showed soft tissue mass throughout the bone cortex. Two lesions were treated with denosumab after four months; CT showed ring-like sclerosis around the outer margin (Figure 4H-4I) and increased sclerosis in the center of the lesion (Figure 4J-4K).

On MRI (*n* = 8), 2 lesions showed marrow replacement by homogeneous tissue, and 6 lesions showed marrow replacement by heterogeneous tissue on T1-weighted images. Two lesions had intermediate signal intensity (similar to that of the muscle), and 3 lesions showed predominately intermediate signal intensity with patchy or stripe-like low signal intensify (Figure 5C); 2 lesions showed mildly higher intensity than muscle, and 1 lesion showed low signal intensity on T1-weighted MR images (Figure 4F). On T2-weighted MR images, the signal intensity was heterogeneous in 6 lesions and homogeneous in 2 lesions. Five lesions showed predominately high signal intensity (similar to that of fat) with patchy or stripe-like low signal intensity (Figure 5D), and 1 lesion showed higher signal intensity than that of fat; 2 lesions showed homogeneous high signal intensity (Figure 4C). One lesion subjected to contrast enhancement showed obvious enhancement. In addition, cortical destruction with an associated soft tissue mass and extensive edema was seen 3 lesions.

PET-CT (*n* = 2) showed increased uptake of ^18^F-PDG, with homogeneity in 5 lesions and heterogeneity in 1 lesion (Figure 3A).

Bone scintigraphy (*n* = 1) showed a diffuse increased radionuclide uptake in the greater trochanter of femur, and a peripherally increased uptake and photopenia centrally in the distal femur (Figure 4A).

### Histologic findings

Seven lesions underwent CT-guided biopsy, and 10 lesions underwent surgical resection. Gross pathology revealed a mixture of reddish-purple soft tissue with yellow areas. Underneath the microscope, 16 lesions showed morphologic characteristics of giant cell tumors of the bone, which demonstrated mononuclear cells and large osteoclast-like giant cells uniformly distributed, and the nuclei of these two cells were indistinguishable. The large osteoclast-like giant cells could have 50-100 nuclei, which displayed a round, “fried-egg” appearance with centrally located nuclei and a wide, thin peripheral rim of acidophilic cytoplasm. Among these cases, 9 lesions penetrated the bone cortex and invaded peripheral striated muscle, which had some proliferative active mononuclear cells and stromal cells, pathological mitosis and some spindle cell transformation. One lesion in the distal femur showed immature osteoid tissue formation with suspicious sarcomatous transformation (Figure 1C), and 1 lesion located in the left forearm, which developed in the blood vessel and penetrated the vascular wall with involvement of peripheral soft tissue, was a giant cell tumor of the bone with a malignant transformation component consisting of well-differentiated osteosarcoma (Figure 2D). Four lesions in the foot and distal tibia showed decreased giant cells with rich, proliferative, active spindle cells, reactive bone hyperplasia and massive fibrous tissue proliferation (Figure 6C-6E).

### Follow-up and prognosis

The follow-up data were obtained from the discovery and initial treatment, and the period ranged from 4 month to 15 years; the patients were mostly offered a radiograph of the affected bone to seek lesion recurrence every three months and chest CT to seek lung metastasis every half year. Three lesions reoccurred. The lesion located in the greater trochanter of the left femur recurred after 4 years from the first treatment, and the interval between the first and second lesions and between the second and third lesions were 4 years and 5 years, respectively (Figure 1). The left distal radius lesion recurred two years after the initial treatment, and another lesion recurred in the left forearm soft tissue (Figure 2). Multi-centric lesions were found in the foot eight years after initial treatment for the left distal tibia lesion (Figure 6). The patient with Paget's disease underwent PET-CT every half year; there was no evidence of local recurrence or formation of another lesion (Figure 5). Three patients were treated with denosumab for one year, a half year and four months, respectively (Figure 1, Figure 3, Figure 4). Currently, all of the patients are in stable condition, no recurrence or new lesions have been detected, and chest CT has shown no evidence of lung metastasis.

## DISCUSSION

In this study, we speculated that MGCT had an increased risk of aggressive behavior based on imaging features, histological characteristics and age at initial presentation. In addition, we found that the greater trochanter of the femur may be another site frequently involved by MGCT, along with the small bones of the hands and feet.

The classical radiographic appearance of GCT is an eccentric, geographic, lucent lesion with a narrow zone of transition but no sclerotic margin. In our study, however, we found 12 lesions that showed ring-like or patchy sclerosis and only 4 that showed typical appearances. These radiographic features were rare in conventional GCT. Helms noted that, if GCT occurred in an epiphyseal-equivalent region, such as the flat bones or an apophasis, the lesion was less likely to have classic features [24]. In addition, Hoch et al noted that a small amount of MGCT could show sclerosis and mineralization in a detailed clinic-pathological evaluation [9]. This was confirmed by Dhillon, who suggested that sclerosis in MGCT correlated histopathologically with abundant reactive bone formation, indicating a fibro-osseous or bone-forming tumor [8]. Spindle-shaped stromal cells are the authentically neoplastic component of the lesions, and the cells themselves are a heterogeneous population that can comprise cells at multiple stages of differentiation [25]. Moreover, one study reported that the stromal cells were not only differentiable into osteoblasts but also into adipocytes and chondrocytes [26]. Cultured stromal cells had the ability to form mineralized nodules, and they could stimulate bone formation when implanted subcutaneously in mouse models [27]. This might explain why GCT shows a sclerotic rim or patchy sclerosis on imaging. Whether the radiographic characteristics are a sign of MGCT or not has not been confirmed.

Moreover, we found 5 lesions with soft tissue masses throughout the bone cortex, one initial lesion with pathological fractures and 8 lesions with cortical discontinuity. On MRI, 3 lesions showed soft tissue masses with extensive edema, which proved to be a prognostic marker of local recurrence [28]. Further, pathological fractures were considered not only a potential marker of aggression [29], but also a prognostic factor for local recurrence [30]. Although the prognostic value of cortical discontinuity and soft tissue mass is still debated, there is broad agreement that soft tissue mass extension definitely influences ideal surgical treatment selection. The prognosis of the patient was closely associated with treatment selection, which might suggest that MGCT has a greater risk of aggressiveness.

There was generally no difference in histopathology between GCT and MGCT [8]. However, we found 9 lesions with some proliferative, active mononuclear cells and stromal cells, pathological mitosis and some spindle cell transformation in our study. Among these cases, one lesion showed malignant transformation consisting of well-differentiated osteosarcoma. If an atypical mitotic figure is detected, it could suggest malignant transformation [31]. Although we did not discover definitive malignant areas, these lesions were suspected to have malignant transformation. Over time, they could become diagnostic for malignant GCT. Peimer et al also noted that MGCT tended to exhibit much more spindle cell transformation than solitary GCT in their series of 5 cases [32]. Our results are similar to their findings. Although histological grading was not predictive of clinical outcome or aggressiveness [33], we believe that if a lesion has features of sarcomatous transformation on pathology, there is likely a greater risk of aggressive behavior. Moreover, GCT was classified into intermediate (rarely metastasized) and malignant biological behaviors in 2013 [2]. The frequency of GCT with malignant transformation was less than 1% and was associated with dedifferentiation of the primary tumor or was secondary to prior radiation therapy[34]. In our study, no patients underwent radiation therapy, but the 16.67% (1/6) rate of malignant transformation was higher than previously reported, which might suggest increased risk of malignant MGCT.

Eighty percent of GCT cases occur within the age range of 20-50 years, with peak incidence at 30 years of age. In our cases, the average age at initial presentation was 22.33±4.54 years, and 3 patients were younger than 20 years old. Kivioja et al. reported that younger patients had an increased risk of local recurrence [35], which could be related to increased bone turnover in young people [35]. Among our cases, the initial lesions recurred in 3 patients, which might also support this point of view.

We also found that MGCT had a tendency to occur in uncommon sites, such as an apophasis, the feet and flat bones in our study. This result was in partial agreement with reports that MGCT occurred more frequently in the small bones of the hands and feet [19, 36]. However, we assumed that when the lesion was located in the greater trochanter or head of the femur, it would alert the doctor to be aware of MGCT. More interestingly, all of the lesions in each patient occurred in the ipsilateral extremity in our study, which might indicate that doctors should emphasize the ipsilateral extremity, especially during follow-up.

With regard to GCT treated with denosumab, the first phase II study demonstrated lesion response in 30 of the 35 patients [12]. Chawla et al reported that among patients administered denosumab, 96% with unresectable GCT did not exhibit lesion progression, 74% with resectable GCTB did not undergo surgery, and 62% who underwent surgery had less complicated procedures than anticipated [37]. In our study, 7 lesions responded to denosumab, among which 6 showed ring-like sclerosis around the outer margin or increased sclerosis in the center of the lesion after four months. One lesion exhibited shrinkage of the osteolytic zone and the formation of sclerosis in the center of the lesion and adjoining bone cortex after one year, which is similar to the result reported by Hakozaki [38]. Because the long-term prognosis with denosumab treatment is not yet clear, the lesion should be carefully followed up.

To attain a correct diagnosis of MGCT, it is necessary to exclude other causes of multifocal osteolytic lesions. The main differential diagnosis includes multiple brown tumors, giant cell reparative granuloma, metastasis, multiple myeloma, Paget's disease, and some primary osseous tumors, together with a giant cell constituent, likely fibrosarcoma, osteosarcoma, angiosarcoma or chondroblastoma. Clinical, radiographic and histological grounds are needed.

There were several limitations of our study. First, we collected only a small number of cases of MGCT because its frequency is very low. Second, not all of the lesions in each MGCT case simultaneously underwent radiography, CT and MR, so we could not directly compare imaging features among multiple modalities and could not directly correlate the imaging features with the pathologic findings. Third, because 2 of the investigators knew that all of the study patients had a presumptive diagnosis of MGCT and evaluation was based on consensus, there was the possibility of inter-reader bias. Fourth, the pathogenetic mechanism of MGCT is not clear, but we were able to determine whether the MGCT was a metastatic disease based on reoccurrence history. Fifth, not all of the patients with MGCT underwent initial testing for ALP, LDH, calcium, magnesium, phosphorus, and no patients were tested serum acid phosphatase or Creatine Kinase iseoenzyme BB (CK-BB). Sixth, the follow-up duration was short, and the patient population treated with denosumab was small, so the long-term prognoses of the patients are not clear. Despite these limitations, we believe that our results provide useful information for more accurate recognition of MGCT.

## CONCLUSIONS

In addition to the small bones of the hands and feet, the greater trochanter of the femur appears to be another frequently involved site of MGCT. When GCT occurs in younger patients, diaphyseal presentation in the small bones (hands and feet) or at uncommon sites with sclerosis, or when GCT is associated with Paget's disease, we should suspect MGCT. CT-scans are better for defining the intraosseous expansion, whereas MRI is better for detecting soft tissue involvement. Because MGCT may have increased risk of aggressive behavior, patients should be carefully monitored for recurrence or new lesion formation, especially in the ipsilateral extremities. Denosumab shows potential efficacy for the treatment unresectable MGCT.

## MATERIALS AND METHODS

### Patients

Our retrospective study was approved by the hospital institutional review board (Shanghai No. 6 People's Hospital), and informed patient consent was not required. We retrospectively reviewed all of the pathologic records of GCT from our institution between 2001 and 2015, and 567 patients were identified with pathologically proved GCT. Among these patients, 6 patients suffered from MGCT. Our inclusion criteria are discussed below: all individual lesions of MGCT were confirmed pathologically. The six patients were treated and followed up in the Department of Orthopedic Oncology.

### Clinical information

The clinical characteristics recorded included patient age, sex, chief complaint (pain, swelling, muscular hypotrophy, limpness, possible soft-tissue mass-decreased joint motion, severe stiffness and pathological fracture), involved anatomic sites-routine blood tests, treatment, and recurrence, as well as a follow-up survey.

### Imaging modality

Radiography was performed on a DR (Siemens, Germany), and all of the patients underwent standard radiographic examinations. CT scans of the patients were performed on a 16-row CT scanner (Siemens Medical Solutions, Erlangen, Germany) and 64-row CT scanner (Light Speed VCT; GE Healthcare, Waukesha, WI) with both bone and soft tissue windows at 2-mm thicknesses and sagittal and coronal reformatted images. MR imaging was performed on 3.0-T superconducting MR scanner (Koninklijke Philips NV, Amsterdam, the Netherlands). The MR imaging sequences included T1-weighted sequences, T2-weighted sequences with and without fat suppression and short-tau inversion recovery (STIR). The scan parameters were as follows: T1W: TR/TE, 400-560/10-20 milliseconds, T2W: TR/TE 1500-3800/70-100 milliseconds; STIR: TR/TE 3000-4000/41.3-48 milliseconds; and TI: 150 milliseconds. The scan planes of the patients included the coronal, sagittal and transverse planes. Some patients underwent MRI contrast enhancement after injection of 0.1 mmol/kg Gd-DTPA into the median cubital vein. The coronal, sagittal and transverse planes of T1-weighted images were performed, and the scan parameters were identical to the those described above. Bone scintigraphy was performed on the SPECT system (Siemens Medical Solutions USA, Inc. Valley Stream Parkway, Malvern, PA) before surgery. Tc-99m methylene diphosphonate was intravenously injected in the patients immediately after preparation. The whole body screening was performed on the Biogram 64 PET/CT system (Siemens Medical Solutions, Germany) before surgery. ^18^F-FDG was intravenously injected in the patients immediately after preparation.

### Analytical methods and observed indicators

All of the imaging findings of the patients, including radiography, CT, MRI, and PET-CT, were reviewed by two musculoskeletal radiologists who had 20 years of image diagnostic experience and complete knowledge of this tumor (Weiwu Yao, Mei li). If the diagnostic outcome was different between the two observers, agreement was reached by consultation. The observer indicators comprised: anatomical location, well-defined or ill-defined; cortical continuity, soft tissue or not; marginal sclerosis or not, homogeneous or heterogeneous, and eccentric or concentric; fluid-fluid level or not; mineralization in the lesion or not; soft tissue-adjoining lesion edema or not; the pattern and degree of enhancement; and increase intake or not.

### Histopathologic analysis

All of the lesions in the six patients who underwent CT-guided biopsy or surgical resection were reviewed by a pathologist with 20 years of experience (Huizhen Zhang).

### Statistical analysis

Quantitative variables are reported as the mean ± standard deviation (SD).
